# Dietary Patterns and Association with Obesity of Children Aged 6–17 Years in Medium and Small Cities in China: Findings from the CNHS 2010–2012

**DOI:** 10.3390/nu11010003

**Published:** 2018-12-20

**Authors:** Dan Liu, Li-Yun Zhao, Dong-Mei Yu, La-Hong Ju, Jian Zhang, Jing-Zhong Wang, Wen-Hua Zhao

**Affiliations:** National Institute for Nutrition and Health, Chinese Center for Disease Control and Prevention, 27 Nanwei Road, Xicheng District, Beijing 100050, China; liudanjulie@163.com (D.L.); zhaoly@ninh.chinacdc.cn (L.-Y.Z.); yudm@ninh.chinacdc.cn (D.-M.Y.); julh@ninh.chinacdc.cn (L.-H.J.); zhangjian@ninh.chinacdc.cn (J.Z.); wangjz@ninh.chinacdc.cn (J.-Z.W.)

**Keywords:** dietary patterns, childhood, obesity, factor analysis, China

## Abstract

Childhood obesity is associated with both near- and longer-term health implications. Few studies have been conducted to explore the associations between dietary patterns and obesity among Chinese children and adolescents. The present study was designed to identify dietary patterns and their relationships with childhood obesity in medium and small cities. This is a cross-sectional study of children participants aged 6–17 years old in the 2010–2012 China Nutrition and Health Survey (CNHS). Socio-demographics, life-style, physical activity, anthropometric variables, and hundred-item food frequency questionnaires (FFQs) were collected. Household income was classified as low, middle, and high. Traffic tools, from non-advanced to advanced, included walking, biking, bus, and car. Dietary patterns were identified using factor analysis of data from FFQs. Two dietary patterns were identified: a Westernized pattern (i.e., high cakes, snacks, sugary beverages, aquatic products, red meat, fruits, and nuts) and a Traditional Chinese pattern (i.e., high cereals, tubers, legumes, fried cereal food, and vegetables). The Westernized pattern was positively correlated with energy intake, household income, traffic tools, and negative correlated with age and housework time. The Traditional Chinese pattern was positively correlated with age, energy intake, and housework time, and negatively correlated with household income and traffic tools. After adjusting for confounding factors, the Westernized pattern was found to be associated with BMI increment, yielding β coefficients (95% confidence interval, 95% CI) of 0.57 (0.40, 0.85) for the fourth quartile. In addition, the Westernized pattern was also found to be significantly associated with an increased risk of obesity, yielding an odds ratio (OR, 95% CI) of 1.49 (1.21, 1.84) from fully-adjusted confounders. Promoting healthier eating patterns could help to prevent obesity in Chinese children. The findings of this study could be used to guide the development of evidence-based preventive nutrition interventions to curb childhood obesity epidemic in small–medium cities in China.

## 1. Introduction 

The prevalence of childhood overweightness and obesity has increased rapidly worldwide in less than one generation [1]. Recent reports have suggested that obesity prevalence may have reached a plateau in some developed countries [2,3], while, in many developing countries, the problems have become far more complex and intractable. Childhood obesity in large cities has tended to grow steadily in China. However, the trend of obesity in small- and medium-sized cities has grown rapidly, with rates varying from 3.2% in 2002 to 7.6% in 2012 [4]. The increase of obesity rates is the most profound in small- and medium-sized cities, compared with that of big cities and rural areas.

Obesity is a complex, multifactorial condition. The vast majority of studies have examined and reported the association between adiposity and environmental and behavioral factors, including diet, lifestyle, and physical activity [5]. Among all the behavioral factors, poor diet patterns in childhood are suggested to be prospectively associated with an increased risk of childhood obesity [6,7]. Intake of free sugars or sugar-sweetened beverages was a determinant of body weight, which became a focus of relevant research [8]. Being the preferred snacking options, sugar-sweetened beverages and sugary snacks are the greatest source of added sugars and have contributed a significant amount of calories to the diets of Chinese children. Although dietary factors have been implicated in the development of obesity, this relationship is complex and not fully understood [9]. Studies focusing on a single food as a risk factor for obesity cannot provide consistent evidence on the associations between dietary factors and obesity [10]. 

To overcome the limitations of the traditional methods of examining single foods or nutrients, dietary pattern analysis was proposed as an approach that examines the joint effects of multiple dietary components [10]. The derivation of dietary patterns to describe diet, as opposed to investigating individual foods or nutrients, has become popular in nutritional epidemiology. Dietary patterns represent the combined effects of foods and illustrate efficaciously the impact of diet on health outcomes [11]. Dietary intakes during childhood may have many short- and long-term impacts on human health, including obesity risks during childhood [12]. The tracking of obesity from childhood into adulthood showed that eating habits developed during childhood may eventually become lifelong dietary habits. 

It has been suggested that Western dietary patterns, characterized by high-sugar and high-fat foods, are associated with higher obesity risk in adolescents [12,13,14]. However, it should be noted that these studies have been mostly conducted in high-income countries and such findings may not be directly applicable to low- and middle-income countries, given the context-specific nature of dietary patterns. The situation in China, which as a rapidly developing country, could be more complicated, as a result of dramatic changes in dietary patterns and lifestyles in the last 20 to 30 years. We aimed to determine the influence of specific dietary patterns on overweightness and obesity in Chinese children and adolescents. The present study explored the association between dietary patterns and obesity among Chinese juveniles in small- and medium-sized cities, where the obesity rate has increased significantly. In this study, dietary patterns were identified using factor analysis. By providing further insight into diet-obesity associations in children and adolescents, the findings of this study could be used to promote the development of effective interventions and policies aimed at curbing the childhood obesity epidemic in China.

## 2. Participants and Methods 

### 2.1. Study Design 

Cross-sectional data from the China Nutrition and Health Survey (CNHS, 2010–2012), which was designed to examine the situation of Chinese nutrition and chronic diseases [15], was used in this study. The CNHS covers 31 provinces that vary in demography, geography, and economic development. A stratified multi-stage cluster sampling method was conducted at 150 survey sites of 4 types: 34 big cities, 41 medium and small cities, 45 general rural areas, and 30 poor rural areas (these types were defined according to economic and social development characteristics). The survey was carried out on a nationally representative sample of China. Households, the primary sampling units in this survey, were drawn using a stratified cluster random sampling frame. 

### 2.2. Study Participants

A total of 8212 participants with socio-demographic indicators, food frequency questionnaires (FFQs), and anthropometric measurements data was recruited in medium and small cities from Chinese children and adolescents aged 6–17 years. Based on the deletion of extreme values, 7988 participants were analyzed in this study.

All the procedures involving human subjects/patients were approved by the National Institute of Nutrition and Health, China Centre for Disease Control and Prevention (Ethic Committee approval code: 2013-018). All participants/parents gave their written informed consent.

### 2.3. Data Collection

In face-to-face interviews, data were collected from participants by trained investigators using age-specific multi-component questionnaires covering information on demographics, socio-economics, and lifestyle characteristics. 

Socio-demographic indicators included the following variables: age, sex, address, and household income, et al. Household income was classified as low, middle, and high level. Lifestyle characteristics included physical activity time in school per day, sedentary time in spare per day, housework time in home per day, and main transport tools in the last three months. Traffic tools, from non-advanced to advanced, included walking, bike, bus, and car. Housework time, sedentary time, and physical activity time were continuous variables. 

Anthropometric measurements included body weight and height. These indicators were taken using standardized techniques and calibrated equipment in all survey sites. Heights were measured once with barefoot and body weight was measured once in light clothes. The accuracy of the height and weight was 0.1 cm and 0.1 kg, respectively. The measurement was carried out in accordance with the unified method, and all data collectors took the unified training and examination, and held the qualification certificate. Each measurement indicator results of each data collector was retested partly to ensure the inter-rater reliability. Each indicator was equipped with two data collectors to prevent errors. Standardized tests were developed by measurement professionals with help of mathematical methods to control errors. Height and weight were used to calculate body mass index (BMI), by dividing weight (kg) by height squared (m^2^). The BMI z-scores were calculated using Chinese standards, age, and gender specifics [16]. 

The dietary intake of participants was assessed using two methods, which were 3 day 24-h dietary recalls and a semi-quantitative FFQ. Dietary intake was assessed using a semi-quantitative FFQ and 3 day 24-h dietary recalls. All questionnaires were designed by a panel of experts, including scientists in the fields of epidemiology and nutrition, and were tested on a convenience sample to check for clarity and cultural sensitivity. Dietary intake data obtained from the FFQ was believed to be more reflective of long-term intake, and therefore, was used for the assessment of dietary patterns [17]. The FFQ referred to dietary intake of the previous year and consisted of one hundred items of foods commonly consumed in China [18], including five frequency categories which were: (1) almost never eat or drink; (2) how many times per day; (3) how many times per week; (4) how many times per month; (5) how many times per year. The dietary intake of each food item was estimated by the participants with the help of investigators and food size reference photographs. The frequency of each food item was then converted to a daily intake. The daily energy consumption of participants was computed using the China food composition database. 

### 2.4. Dietary Assessment

According to the similarity of ingredients, nutrient profile, and/or culinary usage among the foods and the grouping scheme used in other studies, the one hundred food items was reduced into 19 predefined food groups (Table A1). For the food to add up, food intake was converted according to the protein or carbohydrate content of each food before item reduction. For example, the consumption of soybean milk, tofu, and other soybeans products were converted into consumption of soybean according to their content of protein, and the consumption of milk powder was converted into the consumption of milk according to the protein content. Then, for the 19 food groups, the total consumption was determined by summing up the daily portion intake of each item in its group. Exploratory factor analysis was used to extract dietary patterns based on the predefined 19 food groups; factor loadings were based on the dietary intake. The factors were rotated with varimax rotation to simplify the interpretation. As diagnostics for the factor analysis, the correlation matrix between the 19 food groups was visually and statistically examined. This matrix showed a significant χ^2^ (*p* < 0.001) for the Bartlett’s test of sphericity and a Kaiser–Meyer–Olkin test >0.8, indicating that the correlation among the variables was sufficiently strong for a factor analysis. In considering the number of factors to retain, eigenvalues (>1), scree plots, and the interpretability of the factors were evaluated to determine the factors that could be used to describe distinct food patterns. From these analyses, the four-factor solution was selected. Food groups with positive loadings contributed to the dietary pattern, whereas those with negative loadings were inversely associated with the dietary pattern. Food groups with absolute factor loading coefficients of 0.3 and above were considered to be strongly associated within a pattern [19]. Factor scores were calculated by multiple regression method, and each participant received a factor score for each dietary pattern. These scores indicated the degree to which each participant’s diet corresponded to the identified pattern. Quartiles were categorized across the score of each dietary pattern based on the distribution in the whole population, and used for comparison of energy intake, life-style, physical activity factors, and so on.

### 2.5. Statistical Analysis 

Data were described as *n* (%) for categorical variables, including sex, household income, and transport tools; and described as mean and standard deviation(SD) for continuous variables, including age, BMI, housework time, and physical activity time. Participant characteristics by overweight and obesity status were compared by analysis of variance for continuous variables and by chi-square tests for categorical variables. Two dietary patterns were derived using factor analysis with a principal component method. For each dietary pattern identified, participants were categorized into quartiles according to the standardized dietary pattern score. The characteristics by quartiles were expressed as mean for two dietary patterns. Linear trend analysis was conducted by general linear model or the Cochran–Mantel–Haensel statistics for continuous and categorical variables, respectively, to investigate associations of characteristics with factor scores of each dietary pattern. Logistic regression was used to analyze associations of dietary patterns with childhood obesity, in which the lowest quartile was defined as the reference group. Odds ratio (OR) and 95% CI were calculated, and linear trends of ORs were estimated. An unadjusted model and two models adjusted for covariates were fit for each dietary pattern: model 1 was adjusted for age and sex; model 2 was adjusted additionally for household income per family number, total energy intake, housework time, physical activity time, sedentary time, and traffic tools. Multiple linear regression analyses were used to estimate the β coefficient and 95% CI of the association of children’s BMI with dietary patterns scores. Tests for linearity (tolerance > 0.4) of the covariates included in the regression models were performed. Normality of the residuals was assessed using the histogram of standardized residuals and normal probability plot in all regression models. The SAS version 9.4 (SAS Institute Inc., Cary, NC, USA) was used for all computations, and a *p*-value < 0.05 was considered to be significant, and all *p*-values were two-sided.

## 3. Results 

The sample of children and adolescents considered for this study included 3999 male and 3989 female participants. Sample characteristics are shown in Table 1 by overweight and obesity status. Compared to those who were not obese, those participants who were male, younger, not exercise, with advanced traffic tools, shorter housework time, and longer sedentary time were more likely to be obese (*p* < 0.01 or 0.05). 

Factor analysis revealed two main dietary patterns, which together explained 28.9% of the total variance in dietary intake, with the largest variance being explained by the Westernized patterns (21.6%). Factor loadings and the variance explained by each pattern are shown in Table 2. The two patterns were called “Western”, and “Traditional Chinese”. The naming of these patterns was based on the highest food group loadings. The Westernized pattern (Eigenvalue = 4.1) was loaded heavily on cakes, snacks, sugary beverages, aquatic products, red meat, fruits, and nuts. The Traditional Chinese pattern (Eigenvalue = 1.4) was characterized by a high intake of cereals, tubers, legumes, fried cereal food, and vegetables. The intake of 19 food groups across the quartiles of two dietary patterns in Chinese children is shown in Table A2. 

The relationship between socio-demographics and lifestyle characteristics and dietary patterns score quartiles in a nationally representative sample of Chinese children and adolescents are shown in Table 3. The Westernized pattern was positively correlated with energy intake, household income, traffic tools, and negative correlated with age and housework time. The Traditional Chinese pattern was positively correlated with age, energy intake, and housework time, and negatively correlated with household income and traffic tools.

Table 4 shows the results of multivariate linear regression models with children’s BMI. The second and forth quartile of the Westernized pattern was associated with BMI increment, yielding β coefficients (95% CI) from fully-adjusted confounders of 0.63 (0.40, 0.85). There was no significant difference in BMI with the traditional Chinese pattern and frugality pattern. 

Logistic regression analyses results of the association between the two dietary patterns and obesity are shown in Table 5. After adjusting for age, sex, household income per family number, total energy intake, sedentary time, traffic tools, housework time, physical activity time, children in the higher quartiles of the Westernized pattern score were more likely to be obese, and the adjusted OR (95% CI) was 1.49 (1.21, 1.84) for the fourth quartile. 

## 4. Discussion 

In this cross-sectional study, we identified two distinct dietary patterns: Westernized pattern and Traditional Chinese pattern. The present study found that the Westernized pattern was positively associated with the increased risks of childhood obesity after adjustments for putative risk factors. These results suggested that such dietary patterns were independently associated with obesity of children and adolescents in China. We believe that the present study is the first evidence of the relationships between childhood obesity and dietary patterns in a national sample of medium and small cities of China, in which the results were analyzed using food frequency questionnaires (FFQ). 

Previous studies showed that intake of saturated fat, free sugars or sugar-sweetened beverages were associated with high BMI and waist circumference [20,21]. In this study, individuals who adopted a Westernized pattern, which is characterized by higher intake of sugary beverages, snacks, cakes, and red meat, had an increased risk of having obesity. From the end of the last century, Chinese diets changed vastly. Children tended to drink sugar-sweetened beverages, eat sweet cookies, processed meat, and fast food [22,23], which may lead to obesity and other non-communicable diseases (NCDs) if consumption is excessive [24]. In Table A2, we can see the higher intake of sugary beverages, sweets and cakes, red meat, and juice in Westernized pattern for the highest quartile. Therefore, a marked transition to the Westernized pattern has occurred in China, and Chinese teens are experiencing a dramatic increase in snacking, soft drinking, and fast-foods eating [25]. A further study will be carried out to focus on the collection of data on beverages, snacks, and fast-foods.

Several studies have indicated that dietary patterns such as western eating patterns with increased consumption of soft drinks, sweets, snacks, and meat might be positively associated with obesity, although the results have been inconsistent [26]. In our study, we have identified a correlation between Westernized patterns and obesity, which was consistent with most studies [27,28]. It is well accepted that energy consumed in beverage form does not produce the same sensation of satiety as energy from solid food, and therefore, beverages intake will not reduce the intake of other foods [29]. In our study, we found that the intake of beverages, sweets, and cakes was without acceptable limits in Westernized pattern for the fourth quartile. Meanwhile, sugary-rich food intake could be associated with other obesity-related dietary factors, such as saturated fat. In the present study, children who consumed more sugary foods also consumed more red meat and processed meat. Therefore, sugar-fat rich foods were associated with childhood obesity. 

The traditional Chinese pattern characterized high intake of cereals, tubers, legumes, fried cereal food, and vegetables. The results showed that children who had a lower socioeconomic status (SES) including lower household income tended to this dietary pattern. Those children usually purchased affordable fried cereal food instead of meat to satisfy their taste. Socioeconomic status is reportedly inversely associated with obesity in high-income countries [30]. However, the condition was different in developing countries. Our previous studies have confirmed that children with higher SES tended to be obese in China [31]. From the perspective of this study, the cause of obesity in some high SES children may be related to being able to afford high-calorie foods and avoid physical labor, whereas low SES limits the resources available for excess food consumption and increases physically demanding labor.

There is a fact that cannot be ignored. Current persistence of obesity might differ from previous generations, when the prevalence of obesity was lower, and the environment was less “obesogenic” as it is nowadays. Obesogenic environments include the change of dietary patterns and eating behavior, increments in production supply and static activities, social-economic, cultural, and other factors. This leaves a question that it might be difficult to control obesity through interventions to modify environmental factors. Everyone inevitably consumes foods that tend to cause obesity. Our intervention strategy can only reduce the intake of these “obesity” foods rather than eliminate them. Therefore, if the child was inclined to the Westernized pattern, our strategy suggests they avoid excessive intake as well as cultivate a healthy lifestyle. Creating a negative energy balance by decreasing caloric consumption and increasing physical activity is a common strategy used to prevent obesity. For children at school, their time was mostly arranged by the school, and there was no significant difference among school children in physical activity and sedentary time. While, physical activity outside the school was very different. Our study showed that traffic tools were positively related to obesity, and housework was negatively related to obesity. Therefore, there is a need to promote leisure-time physical activity, such as walking or riding to school instead of by car and active housework at home.

Given the key roles of social and environmental factors in shaping dietary habits, population-based approaches are crucial to achieve broad success in personal behavioral change. Firstly, participants in the catering market or food industry, from food manufacturers to retailers and restaurateurs, must commit to providing healthier food. Secondly, social media must promote consumer education and should have the right guidance for children. Thirdly, appropriate policies may also provide promising opportunities to reduce the adverse health impacts of poor diets. Overall, close collaboration among individuals and multiple stakeholders, including those at the sociocultural, community, agricultural, industrial, and governmental sectors are required. 

Some limitations need to be addressed in the study. First, the cross-sectional design of this study cannot draw conclusions about the etiological link between dietary patterns and obesity. Second, the statistical methods used to identify dietary patterns are somewhat subjective, including the consolidation of food items into food groups, the number of factors to extract, and the labelling of the pattern [32]. Third, the intermediary role between SES and dietary patterns needs to be further studied. However, these limitations do not affect the significance of the study. To our knowledge, this is the first evidence to reveal the relationships between dietary patterns and risk of obesity in Chinese children and adolescents by FFQ among medium- and small-sized cities using nationally representative data. Unlike 24-h recalls, which are considered short-term dietary assessment tools, the FFQ covers a longer period of dietary recall [9,33]. Measuring average long-term diet may be more valuable than measuring the intake of a few specific days, particularly when aiming to assess the relation between food and related long-latent diseases with modifiable risk factors, such as obesity and diet related non-communicable diseases [17]. China is undergoing a rapid economic or nutritional transition. Although the current Chinese guidelines provide general dietary advice to children and adolescents, SES-specific dietary guidelines for teenagers are needed in the prevention of obesity and NCDs [34]. The evidence in our study can inform policy-makers and the developers of programs for preventing overweightness and obesity in Chinese children and adolescents.

## 5. Conclusions

In conclusion, the Westernized pattern was positively associated with obesity among children and adolescents in China and the children are suggested to avoid excessive intake as well as to cultivate a healthy lifestyle. Actions should be taken to promote childhood obesity interventions. Further studies are needed to understand more objectively the relationship between dietary patterns and obesity using prospective data.

## Figures and Tables

**Table 1:** General characteristics of study participants (*n* = 7988).

	Overweight and Obesity				*p*
	No (6391)		Yes (1597)		
Age (years, SD)	12.1	3.3	11.2	3.2	<0.01
6–11 years (*n*, %)	3100	48.5	981	61.4	
12–17 years (*n*, %)	3291	51.5	616	38.6	
Sex (*n*, %)					<0.01
male	3051	47.8	948	59.4	
female	3340	52.3	649	40.6	
BMI (mean, SD)	17.5	2.4	23.0	4.0	<0.01
BMI (media, IQR)	17.2	15.6, 19.2	22.7	20.1, 25.2	
Household income (*n*, %)					>0.05
Low	1852	29.0	425	26.7	
Middle	2243	35.1	546	34.2	
high	842	13.2	242	15.2	
unknown	1453	22.7	382	24.0	
Housework time/d (min, mean, SD)	16.4	18.0	14.5	17.0	<0.01
Sedentary time/d (h, media, IQR)	3.0	2.0, 4.0	3.0	2.0, 3.5	<0.01
Physical activity time/d (min, mean, SD)	63.0	36.4	62.2	33.1	>0.05
Exercise in spare time (*n*, %)					<0.05
Yes	3859	60.4	919	57.6	
No	2526	39.6	677	42.4	
Traffic tools (*n*, %)					<0.01
Walk	2853	44.7	703	44.1	
Ride	925	14.5	189	11.9	
By bus	1061	16.6	235	14.7	
By car	1366	21.4	443	27.8	
Other	185	2.9	25	1.6	

BMI: Body mass index; Data are shown as number (*n*, %) for categorical variables; and shown as mean and standard deviation (SD) for continuous variables; IQR: interquartile range, d: day, h: hour, min: minutes.

**Table 2:** Factor-loading matrix for the two dietary patterns and their food or food groups in Chinese children.

Food or Food Groups	Westernized Pattern	Traditional Chinese Pattern
Wheat and rice	0.20	0.36 *
Other cereals and tubers	−0.05	0.75 *
Fried cereal food	0.09	0.60 *
Legumes	0.11	0.67 *
Vegetables	0.32 *	0.49 *
Salted vegetables	0.04	0.35 *
Fungi and algae	0.31 *	0.33 *
Fruits	0.51 *	0.27
Milk	0.49 *	0.01
Red meat	0.53 *	0.32 *
Processed meat	0.32 *	0.22
Poultry	0.42 *	0.12
Aquatic products	0.54 *	0.19
Eggs	0.42 *	0.02
Nuts	0.50 *	0.15
Cakes and snacks	0.61 *	0.30 *
Fresh fruit and vegetable juice	0.49 *	0.00
Sugary beverages	0.57 *	0.09
Coffee and tea	0.33 *	0.04
% of explained variance	21.6%	7.3%

* Means factor loading with absolute value ≥ 0.3.

**Table 3:** Association of socio-demographics and lifestyle characteristics with dietary patterns in a nationally representative sample of Chinese children and adolescents (β Coefficients and 95% confidence intervals).

	Westernized Pattern		Traditional Chinese Pattern	
	*β*	95% CI	*β*	95% CI
Age (years, SD)	−0.09 **	−0.13, −0.05	0.22 **	0.18, 0.27
Male vs. Female	0.03	−0.01, 0.07	0.05 *	0.01, 0.09
Energy	0.64 **	0.63, 0.66	0.48 **	0.46, 0.50
Household income	0.11 **	0.09, 0.12	−0.08 **	−0.10, −0.06
Physical activity	−0.02	−0.03, 0.00	−0.01	−0.03, 0.01
Traffic tools	0.03 **	0.01, 0.04	−0.07 **	−0.09, −0.06
Housework time	−0.02 *	−0.05, 0.00	0.08 **	0.05, 0.10
Sedentary time	0.02	0.00, 0.04	0.02	0.00, 0.04

Adjusted for age, sex, household income per family number, sedentary time, traffic tools, housework time, and physical activity time. Household income, sedentary time, housework time, and physical activity time were analyzed as quartile variables. * Means *p* < 0.05; ** means *p* < 0.01.

**Table 4:** Multivariate linear regression model to evaluate the effect of dietary pattern scores on BMI in Chinese children and adolescents (β Coefficients and 95% confidence intervals).

	BMI (kg/m^2^)		
	*β*	95% CI	*p*
Westernized pattern			
Q1	0	–	
Q2	0.20	−0.02, 0.42	>0.05
Q3	0.13	−0.07, 0.34	>0.05
Q4	0.57 **	0.40, 0.85	<0.001
Traditional Chinese pattern			
Q1	0	-	
Q2	−0.12	−0.32, 0.08	>0.05
Q3	−0.09	−0.30, 0.11	>0.05
Q4	0.01	−0.20, 0.23	>0.05

Q, quartile. Adjusted for age, sex, household income per family number, total energy intake, sedentary time, traffic tools, housework time, and physical activity time. Household income, sedentary time, housework time, and physical activity time were analyzed as quartile variables. ** means *p* < 0.01.

**Table 5:** Association of dietary patterns with childhood obesity in China (odds ratios and 95% confidence intervals).

	Unadjusted		Model 1		Model 2	
	OR	95% CI	OR		OR	95% CI
Westernized pattern						
Q1	1	-	1	-	1	-
Q2	1.08	0.92, 1.26	1.05	0.89, 1.23	1.08	0.92, 1.27
Q3	0.99	0.84, 1.16	0.98	0.83, 1.15	1.07	0.90, 1.28
Q4	1.19 *	1.02, 1.39	1.26 **	1.08, 1.47	1.49 **	1.21, 1.84
*p* for trend	0.07		0.01		<0.01	
Traditional Chinese pattern						
Q1	1	-	1	-	1	-
Q2	0.84 *	0.72, 0.97	0.86	0.73, 1.00	0.86	0.74, 1.00
Q3	0.82 *	0.70, 0.95	0.87	0.75, 1.02	0.89	0.75, 1.04
Q4	0.84 *	0.72, 0.98	0.99	0.84, 1.16	1.00	0.84, 1.20
*p* for trend	0.03		0.84		0.97	

Q1 was the reference group, a < 0.05, b < 0.01; Model 1: adjusted for age, sex; Model 2: Model 1 additionally adjusted for household income per family number, total energy intake, sedentary time, traffic tools, housework time, physical activity time; household income, sedentary time, housework time, and physical activity time were analyzed as quartile variables. *p* for trend was calculated using generalized linear models for categorical variables. * Means *p* < 0.05, ** means *p* < 0.01.

## Appendix A

**Table A1:** Food groups in the factor analysis.

Food or food groups	Foods included in the group
Wheat and rice	Rice, steamed bread, wheat flour noodles, etc.
Other cereals and tubers	Millet, buckwheat, corn, sweet potato, etc.
Fried cereal food	Fried dough stick, fried cake, fried chips, instant noodles, etc.
Legumes	Soybeans, and products
Vegetables	Cabbage, eggplant, carrot, lettuce, tomato, cauliflower, etc.
Fungi and algae	Mushroom, kelp, laver
Fruits	Berries, citrus, kernel fruit, etc.
Milk	Milk and products
Red meat	Pork, beef, goat, lamb
Meat products	Ham sausage, et al.
Poultry	Chicken, duck, goose
Aquatic products	Fish, shrimp, crab, shellfish
Eggs	eggs
Nuts	Peanuts, walnuts, etc.
Cakes and snacks	Cookies, cakes, candied fruit, chocolates, Ice cream, etc.
Fresh fruit and vegetable juice	Freshly squeezed fruit juices and vegetable juices
Sugary beverages	Carbonated drinks, Prepackaged juice, Sweetened milk beverage, Sweet tea beverage, etc.
Coffee and tea	Coffee, tea

## Appendix B

**Table A2:** Food intakes across the quartiles of two dietary patterns in Chinese children (mean).

Food or Food Groups (g)	Q1	Q2	Q3	Q4	*p* for Trend
**Westernized Pattern**					
Wheat and rice	207.4	213.3	236.0	290.4	<0.001
Other cereals and tubers	40.1	23.8	26.3	35.4	<0.001
Fried cereal food	30.8	24.4	30.1	42.8	<0.001
Legumes	20.3	16.7	21.8	31.6	<0.001
Vegetables	115.8	135.4	168.6	241.7	<0.001
Salted vegetables	4.5	2.4	2.4	4.6	<0.001
Fungi and algae	13.0	19.6	28.3	55.6	<0.001
Fruits	71.3	112.8	156.1	269.8	<0.001
Milk	57.7	128.7	175.0	302.5	<0.001
Red meat	31.9	48.0	72.5	134.0	<0.001
Processed meat	5.5	8.6	13.1	23.3	<0.001
Poultry	4.4	8.2	13.4	30.9	<0.001
Aquatic products	12.6	20.4	35.3	82.6	<0.001
Eggs	19.3	32.5	43.5	55.2	<0.001
Nuts	3.6	5.5	10.3	27.3	<0.001
Cakes and snacks	19.3	33.5	56.7	113.1	<0.001
Fresh fruit and vegetable juice	2.0	4.6	10.1	34.9	<0.001
Sugary beverages	31.7	69.6	114.2	228.7	<0.001
Coffee and tea	0.7	0.8	1.9	10.9	<0.001
**Traditional Chinese pattern**					
Wheat and rice	174.1	216.8	243.2	313.4	<0.001
Other cereals and tubers	8.8	15.4	28.1	73.6	<0.001
Fried cereal food	12.4	19.7	30.9	65.3	<0.001
Legumes	8.6	13.3	19.9	48.8	<0.001
Vegetables	83.2	124.3	167.4	287.0	<0.001
Salted vegetables	0.8	1.4	2.4	9.3	<0.001
Fungi and algae	13.2	18.7	28.3	56.3	<0.001
Fruits	103.3	125.3	153.5	227.4	<0.001
Milk	190.6	147.9	149.3	174.6	<0.001
Red meat	46.9	55.4	72.2	111.8	<0.001
Processed meat	7.0	8.9	12.5	22.1	<0.001
Poultry	11.6	11.8	13.9	19.6	<0.001
Aquatic products	29.3	34.2	35.3	51.9	<0.001
Eggs	39.9	35.7	35.9	38.7	<0.001
Nuts	9.0	8.8	10.6	18.3	<0.001
Cakes and snacks	33.5	43.6	53.9	91.5	<0.001
Fresh fruit and vegetable juice	16.6	9.7	11.2	14.0	<0.001
Sugary beverages	104.4	90.9	109.0	139.1	<0.001
Coffee and tea	3.8	2.7	3.0	4.8	<0.001

Q, quartile; *p* for trend was calculated using generalized linear models for continuous variables. We performed tests for linear trend by entering the median value of each category of dietary pattern as a continuous variable in the models.
